# Mr. Bawden's Munificence

**Published:** 1905-09-16

**Authors:** 


### EDITOR'S LETTER-BOX.

[Our Correspondents are reminded that prolixity is a great bar to publication, and that brevity of style and conciseness of statement greatly facilitate early insertion.]

### MR. BAWDEN'S MUNIFICENCE.

A correspondent writes:—The magnificent gift of £100,000 recently made by Mr. E. G. Bawden, of the Stock Exchange and Clapton, to a number of charitable institutions, and distributed for him by Mr. Edgar Speyer, in accordance with a scheme which has been approved by the donor and widely published, furnishes one of the most remarkable of modern instances of generosity, concerning which it seems almost ungracious to speak in any other language than that of eulogy. It is not impossible, however, that the example thus set may be followed by other wealthy men; and the very fact that Mr. Bawden appears to have distrusted his own judgment in the matter, and to have appealed to Mr. Speyer for advice and assistance, is of itself a sufficient acknowledgment that the allotment of gifts to objects is a task by no means devoid of difficulty, and one which requires a very ample knowledge of the aims and resources of existing institutions before it can be accomplished in a completely satisfactory manner. Perhaps the first question which must always present itself to the mind of a large donor must be whether it is best, on the whole, to divide his donation among many objects, as Mr. Bawden has done, or to concentrate it upon a single one, which, by virtue of that concentration, might be carried out in a completely effectual manner. We apprehend that the latter course is that which would most frequently commend itself to the judgment, even if only because most persons have had some particular necessity or requirement brought prominently before their minds, and are, therefore, as it is said, more "interested" in it than in others which might appear equally important to the absolutely impartial, if such could be discovered among mankind. The attainment of a single object, moreover, would be more likely to appeal to the imagination than the mere promotion of many, however useful or laudable they might be; and it would generally be possible to discover by inquiry some pressing necessity to which money might with propriety be devoted. The *Times*, in commenting upon Mr. Bawden's gifts, pointed out to others who might be disposed to emulate his liberality that one of the great needs of the day is a provision for the cost of research in clinical laboratories, research by which light might be thrown upon many of the more obscure processes of disease, and by which great benefits might thus be conferred upon the whole community. It referred, in this connection, to the recent writings of Dr. Wright, lately professor of pathology at Netley, and now pathologist of St. Mary's Hospital, who has been well known as the advocate of inoculation as a prophylactic against enteric fever, and who withdrew himself from the War Office Commission appointed to inquire into the subject, because he was not satisfied with the conditions under which their inquiry was to be conducted. The stringent requirements of the man of science were unintelligible to the men who would probably describe themselves as "of business," and so the problem to be solved will for the present continue to await solution. Dr. Wright has lately supplied an article on the subject of clinical research to the *Liverpool Daily Post*, presumably in the not unreasonable expectation that a community of merchants which has liberally supported the local School of Tropical Medicine, and is already beginning to experience the value of its work, will be more disposed than others to afford the means of performing analogous work in other directions. It cannot be said that the picture which Dr. Wright has there drawn of the present state of clinical medicine is a flattering one, and there are no doubt many who will regard his account of its deficiencies as exaggerated; but, at the same time, he has certainly called attention to one of the gravest of the wants of our time, and to one which, if the necessary funds were provided, there would be no lack of earnest workers to supply. Dr. Wright specially mentions the absence of any means of effectual interposition, on the part of the physician, in the constantly recurring strife between a human organism and the pathogenetic microbes by which it is liable to be invaded; and he desires to find agencies which may play, in relation to enteric and scarlet fever, or to other zymotic diseases, the part which is now played by quinine in ague, or by anti-toxin in diphtheria. The desire is a perfectly reasonable one, and the clinical laboratory is manifestly the machinery by which it will one day be satisfied.

